# Genome-wide identification of MIKC-type genes related to stamen and gynoecium development in *Liriodendron*

**DOI:** 10.1038/s41598-021-85927-7

**Published:** 2021-03-22

**Authors:** Huanhuan Liu, Lichun Yang, Zhonghua Tu, Shenghua Zhu, Chengge Zhang, Huogen Li

**Affiliations:** 1 College of Forestry, Nanjing Forestry University, Nanjing, 210037 China; 2 Key Laboratory of Forest Genetics and Biotechnology of Ministry of Education, Co-Innovation Center for Sustainable Forestry in Southern China, Nanjing Forestry University, Nanjing, 210037 Jiangsu China

**Keywords:** Developmental biology, Genetics, Molecular biology, Plant sciences

## Abstract

The organogenesis and development of reproductive organs, i.e., stamen and gynoecium, are important floral characteristics that are closely related to pollinators and reproductive fitness. As a genus from Magnoliaceae, *Liriodendron* has only two relict species: *L. chinense* and *L. tulipifera*. Despite the similar flower shapes of these species, their natural seed-setting rates differ significantly, implying interspecies difference in floral organogenesis and development. MADS-box genes, which participate in floral organogenesis and development, remain unexplored in *Liriodendron*. Here, to explore the interspecies difference in floral organogenesis and development and identify MADS-box genes in *Liriodendron*, we examined the stamen and gynoecium primordia of the two *Liriodendron* species by scanning electron microscopy combined with paraffin sectioning, and then collected two types of primordia for RNA-seq. A total of 12 libraries were constructed and 42,268 genes were identified, including 35,269 reference genes and 6,999 new genes. Monoterpenoid biosynthesis was enriched in *L. tulipifera*. Genome-wide analysis of 32 MADS-box genes was conducted, including phylogenetic trees, exon/intron structures, and conserved motif distributions. Twenty-six genes were anchored on 17 scaffolds, and six new genes had no location information. The expression profiles of MIKC-type genes via RT-qPCR acrossing six stamen and gynoecium developmental stages indicates that the *PI-like*, *AG/STK-like*, *SEP-like*, and *SVP-like* genes may contribute to the species-specific differentiation of the organogenesis and development of reproductive organs in *Liriodendron*. Our findings laid the groundwork for the future exploration of the mechanism underlying on the interspecific differences in reproductive organ development and fitness in *Liriodendron*.

## Introduction

Flowers are the reproductive structures of angiosperms, and the stamen and gynoecium morphologies are thought to be the most important characteristics for taxonomy research and plant fertilization. Hence, particular attention has been paid to floral anatomy and morphology in plants. As “basal angiosperms”, Magnoliaceae plants present several ancestral floral traits that are critical for research on floral morphology evolution in these plants. *Liriodendron* (Magnoliaceae) species survived the last ice age and are represented by only two relict species: *L. chinense* Sarg. and *L. tulipifera* L.^1,2^. These species have similar floral morphologies, although the latter has brighter and significantly more colorful flowers with rich nectar^3^. *L. chinense* has a low seed-setting rate at ≤ 10% under natural conditions, and it was listed on the Red List of Endangered Plants in China in 2004^4,5^. Although many studies have focused on the mechanisms underlying the low seed-setting rate in *L. chinense*, consistent conclusions have not been reached^6^. Reproductive organs are obviously important for flowering plants and closely related to the pollinators and seed-setting rate, i.e., plant reproductive fitness. Therefore, reproductive organ development is a vital factor underlying the difference in the seed-setting rate between the two species. Based on morphological and proteomic analyses, Li et al. suggested that the pistil feature might be the main reason for the low seed-setting in *L. chinense*^6^*.* One pistil potentially produces two ovules, and the stigmatic pollen load showed a correlation with the seed-setting rate, but the high stigmatic pollen loads did not always result in a high seed-setting rate^7–9^. The floral syndrome of *L. chinense* seemed to be adaptive to insect pollination, and flies and beetles were recognized as the main flower visitors^8^. Several reports have investigated stamen and gynoecium development in *Liriodendron* species, although these reports mostly focused on the later stages of *L. chinense* development. The early stages of reproductive organ organogenesis and development in *Liriodendron* species, especially the molecular regulation of the associated genes, have not been examined. The early stage of flower development can be divided into three stages: floral induction, floral meristem formation and floral organ primordium formation. After three early stages of development, floral organ primordia grow and develop to form floral organs^10,11^. The early stages of reproductive organ organogenesis and development determine the floral structure. These transitions are promoted by genes identified to encode transcription factors (TF)^12,13^. Therefore, it is vital to study the early stages of reproductive organ organogenesis and development in *Liriodendron* species to explore the differences between *L. chinense* and *L. tulipifera*.

The MADS-box transcription factor family is widely involved in various aspects of development, reproduction, and flower formation in plants^14^. MADS-box genes have been generally recognized to play important roles in floral organ differentiation, flowering time, and fruit development and ripening in angiosperms^15,16^. These genes have a highly conserved MADS domain (for MCM1, AG, DEF and SRF) composed of approximately 55–60 amino acids and are divided into two categories, i.e., type I and type II, according to phylogenetic analysis^15,16^. The number of type-I MADS-box genes is greater than that of type-II MADS-box genes^17^. Type-I (M-type) MADS-box genes are further divided into four groups in plants: Mα, Mβ, Mγ, and Mδ^17,18^. In *Arabidopsis*, Mδ genes can also be designated MIKC-type MADS-box genes based on their close relationships^18^. Compared with type-I MADS-box genes, type-II MADS-box genes have been more widely studied because they are involved in various developmental processes and contain more domains^17^. The type-II MADS-box proteins in plants contain three domains other than the MADS domain: an intervening (I) domain, a keratin-like (K) region, and a C-terminal domain. The K-domain (approximately 70 amino acids) is a conserved region typical of plants, and it is found only in type-II MADS domains^15^. Therefore, the type-II MADS-box genes can also be called MIKC-type genes that are specific to plants. The four domains are essential for dimerization, higher-order complex formation, and transcriptional regulation in plants. In angiosperms, there are nearly 100 MADS-box proteins, and MADS-box protein diversity was considered to be related to the mechanism underlying flower diversification^19,20^. Amplification of MADS-box genes in flowering plants may lead to new functions and alter flower morphology and reproductive organ development^16,17,20^.

The ABCDE model genes, a gene classes found in both gymnosperms and angiosperms, belong to the MIKC-type MADS-box genes. In 1991, the classic ABC model was proposed to explain the genetic programs specifying the identities of floral organs by double-mutant and single-mutant phenotype research, and this model involves three classes of functional genes, i.e., A, B and C functional genes^13,21,22^. A complete loss of A-class genes led to the transformation of sepals into carpels; the loss of B-class genes led to the transformation of sepals into petals and stamens into carpels; and the loss of C-class genes led to the transformation of stamens into petals and carpels into sepals. Later, the ABC model was expanded to the ABCDE model in *Arabidopsis thaliana* and *Antirrhinum majus*. These models specified floral organ identity and showed a framework for understanding homeotic genes in plants^13^. The floral organs were determined by different combinations of A-, B-, C-, D- and E-class genes, i.e., sepals (A + E: *AP1* + *SEP*), petals (A + B + E: *AP1* + *AP3* + *PI* + *SEP*), stamens (B + C + E: *AP3* + *PI* + *AG* + *SEP*), carpels (C + E: *AG* + *SEP*), and ovules (C + D + E: *AG* + *AGL11/STK* + *SEP*), by a series of mutant phenotype studies in *Arabidopsis*^22,23^. In the model, the class-A gene is *APETALA1* (*AP1*), the class-B genes are *PISTILLATA* (*PI*) and *APETALA3* (*AP3*), the class-C gene is *AGAMOUS* (*AG*), the class-D gene is *AGAMOUS-LIKE 11* (*AGL11*) (also known as *SEEDSTICK* (*STK*)), and the class-E genes are *SEPALLATA-like* genes (*SEP1,2,3,4*) in *Arabidopsis*^24–33^. In addition, other subgroups, such as *TOMATO MADSBOX3* (*TM3*), *FLOWERING LOCUS C* (*FLC*), and *SOLANUM TUBEROSUM MADS-BOX 11* (*STMADS11*), have also been identified in many species^17^. Their functions and expression patterns are highly conserved in angiosperms.

An increasing number of MADS-box genes have been identified from *Arabidopsis*, *A. majus*, *Glycine max*, *Zea mays*, *Lactuca sativa*, *Brassica oleracea*, and other species, and their functions and mechanisms of action have been extensively studied. However, there are few reports on MADS-box genes in *Liriodendron* species. With the rapid development of various types of omics, such as metabolomics, proteomics, and transcriptomics, we have more techniques and new insights for exploring the molecular mechanisms of reproductive organogenesis. The *L. chinense* genome (1.75 G), which was successfully sequenced in 2019, is vital for investigations into many gene families and molecular regulatory mechanisms of different organ developmental processes in *Liriodendron* species^34^. In this study, early reproductive organ development was observed by scanning electron microscopy (SEM) and paraffin sectioning to identify the morphological and temporal differences between the two *Liriodendron* species, and then, we selected the stamen and gynoecium primordia for a transcriptomic assay. Subsequently, we identified MADS-box genes from the RNA-seq and genome data and performed a genome-wide analysis of these genes in terms of phylogenetic trees, exon/intron structures, conserved motif distributions, and chromosomal locations. Finally, we examined the tissue-specific and species-specific expression patterns of MIKC-type genes in detail during the different stages of stamen and gynoecium development to identify vital candidate genes.

## Results

### Floral morphology and cytology of reproductive organ primordia in *Liriodendron* species

**Figure 1:**
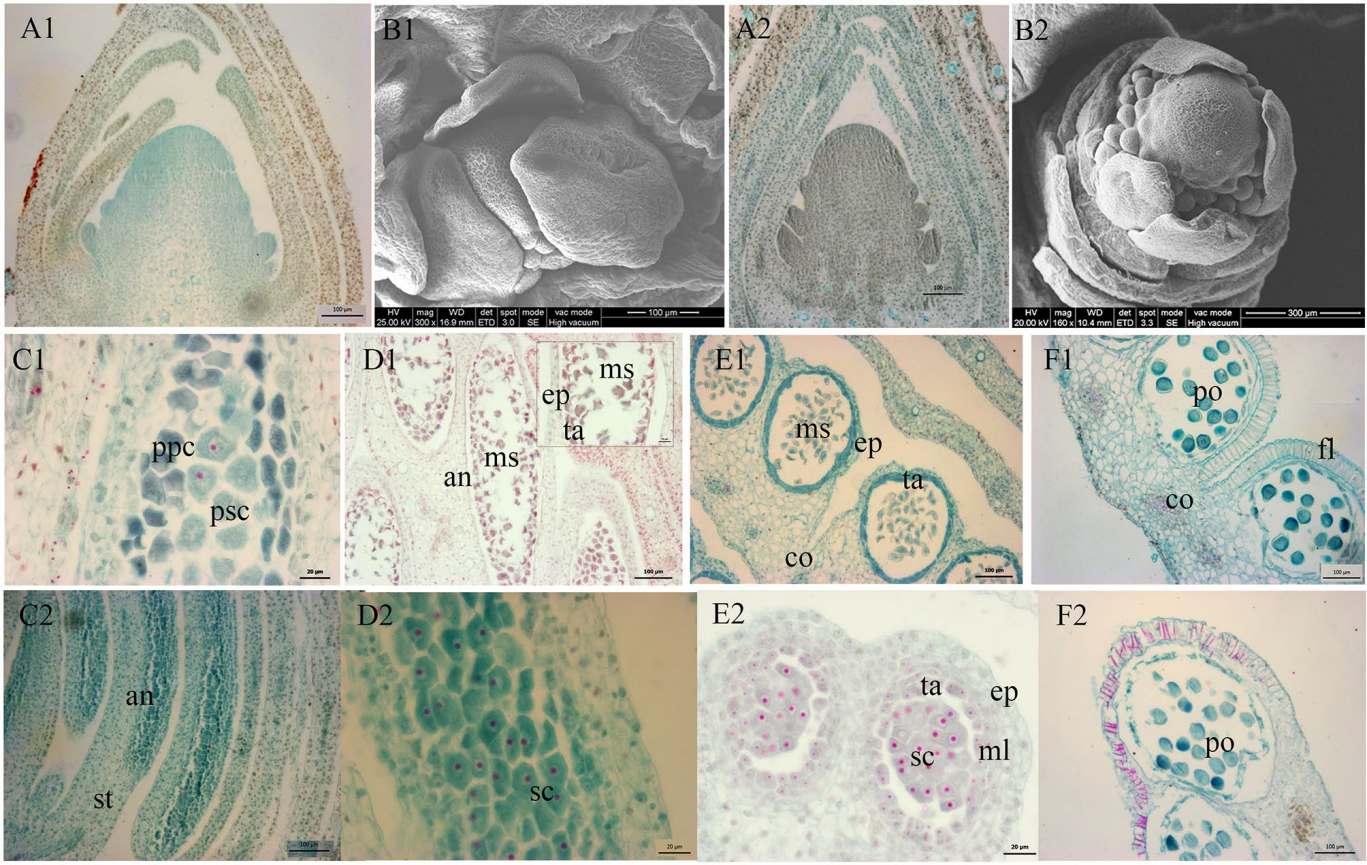
Morphological and cytological observations of the stamen. (**A1–B1**) Stamen primordia of *L. chinense*; (**A2–B2**) Stamen primordia of *L. tulipifera*; (**C1–F1**) Ddevelopment process of anther primordium of *L. chinense*; (**C2–F2**) Development process of anther primordium of *L. tulipifera*; A1, B1, A2, D1, E1, F1, C2, and F2: bar, 100 μm; B2: bar, 300 μm; C1, D2, and E3: bar, 20 μm; *an* anther, *co* connectivum, *ep* epidermis, *fl* fibrous layer, *ml* middle layer, *ms* microsporocyte, *nu* nucellus, *po* pollen, *ppc* primary parietal cell, *psc* primary sporogenous cell, *sc* sporogenous cell; *st* stamen, *ta* tapetum.

**Figure 2:**
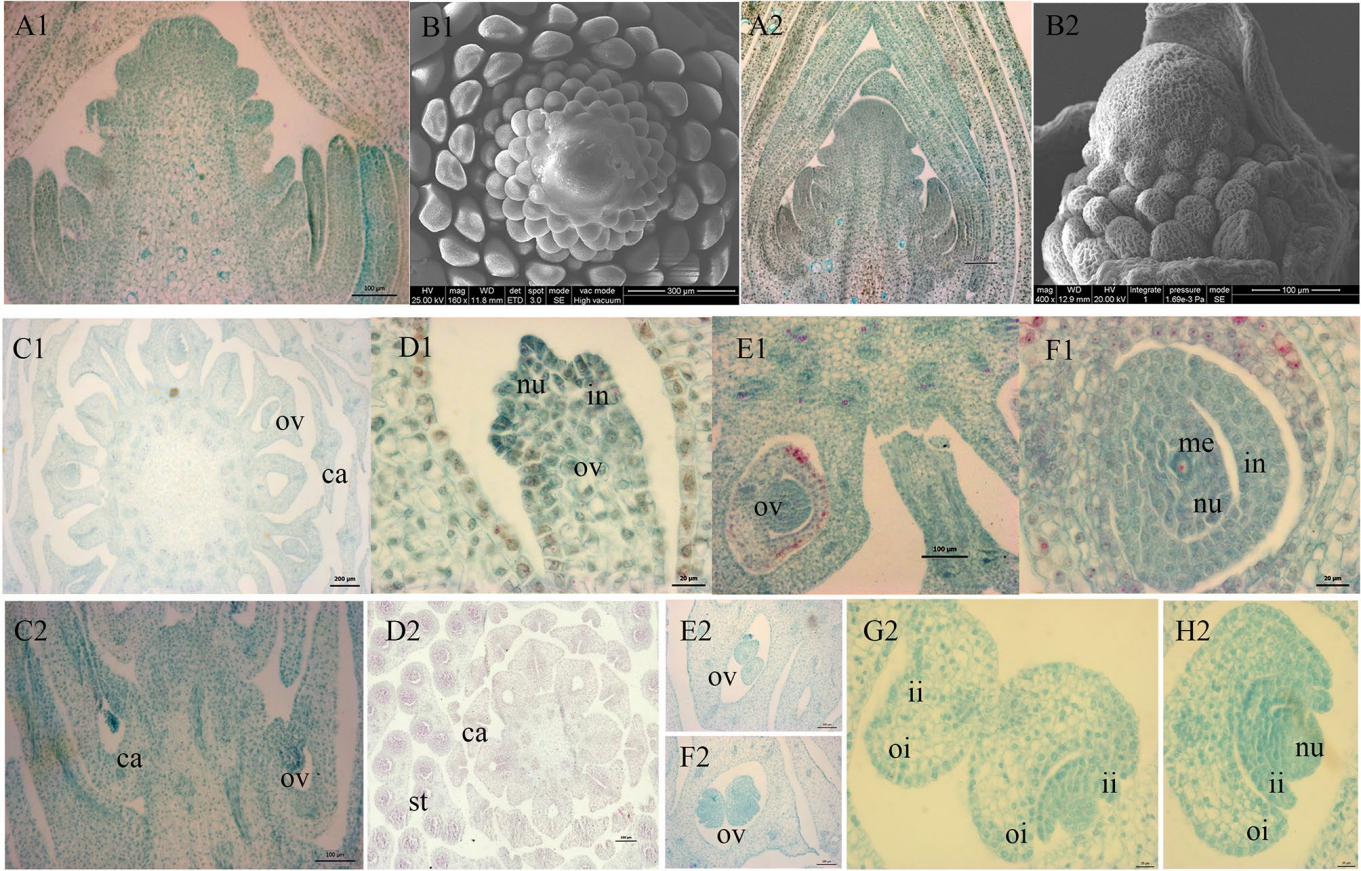
Morphological and cytological observations of the gynoecium. (**A1-B1**) Gynoecium primordia of *L. chinense*; (**A2-B2**) Gynoecium primordia of *L. tulipifera*; (**C1-F1**) Ovule primordia; C2–H2: The two ovules in an ovary; A1, A2, B2, E1, C2, D2, E2, and F2: bar, 100 μm; B1: bar, 300 μm; D1, F1, G2, and H2: bar, 20 μm; C2: bar, 200 μm; *ca* carpel, *ii* inner integument, *in* integument, *me* megasporocyte, *nu* nucellus, *oi* outer integument, *ov* ovule.

The shoot apical meristems of *Liriodendron* species develop into floral meristems less than two months after anthesis, and almost four months are required for the floral organ primordia to arise and differentiate. The first primordia to arise are vegetative organs; then, the stamen primordia and gynoecium primordia appear. The vegetative tissue primordia are three-whorled, although the reproductive tissue primordia arise spirally with centrality. Finally, all carpels fuse into a single gynoecium. The shape and morphology of reproductive tissue primordia are similar between *L. chinense* and *L. tulipifera*. However, the size and number in *L. chinense* are greater than those in *L. tulipifera* (Figs. 1, 2).

The stamen primordia in *L. chinense* are arranged in four whorls with more than 10 organs per whorl. The first stamen primordia of *L. tulipifera* arise in late August. Finally, they form three whorls, with more than 10 organs per whorl. The number of stamens is approximately 38, which is far less than that in *L. chinense*, approximately 59. The filament appears first in mid-February, and the stamen with four anthers wraps around the gynoecium. The anther primordium arises on the abaxial side and develops into primary sporogenous cells with multiple nuclei and primary parietal cells. Subsequently, the anther primordia develop into the anther wall and microsporocytes. In a longitudinal section of the four-locule anther, the organ consists of connectivum, vascular bundle, sporogenous cells, tapetum, middle layer, and epidermis (Fig. 1C–F). At the flowering stage, the length of stamens in *L. chinense* and *L. tulipifera* was 2.69 cm and 3.71 cm, respectively.

The carpel primordia in *L. chinense* develop spirally upward from the stamen base in mid-August. The carpel primordia have nine whorls, with more than 10 organs per whorl. The carpel is initially hemispherical, and then it develops a triangular and imbricate shape (Fig. 2A,B). The carpel primordia in *L. tulipifera* can be seen in early September, which is later than that in *L. chinense*, and have approximately six whorls in total, which is less than that in *L. chinense*. According to these results, we can confirm when the stamen primordia and gynoecium primordia appear and then collect them for RNA-seq analysis.

In late February, dormancy is broken, and the carpels begin to curl from both sides. Subsequently, the ovule primordium arises and develops into the anatropous ovule. Each ovary has two ovules, and each ovule has two integuments. The outer and inner integuments arise around the nucellus almost simultaneously. At this point, the nucellus primordium arises, with the integument primordium on both sides. The spherical nucellus and megasporocyte appear last (Fig. 2C–H). The shape and size of the ovary differs between the two species, and the frequency of forming two ovules in *L. tulipifera* is higher than that in *L. chinense*. We speculate that *L. chinense* is vulnerable to abortive gynoecia. At flowering stage, the length of gynoecia in *L. chinense* and *L. tulipifera* was 3.63 cm and 4.07 cm, respectively. In brief, these results indicated that the two species in *Liriodendron* share many similar characteristics of reproductive organs, and the differences are mostly in shape and size. Although the size and number of stamens in *L. chinense* are higher than those in *L. tulipifera*, the seed-setting rate of *L. chinense* is significant lower than that of the latter. Accordingly, we infer that the plentiful pollens may be redundant and are not the restrictive reproductive resources in *L. chinense*, and more likely, the low seed-setting rate of *L. chinense* is due to its abortive gynoecia.

### Sequencing, assembly, and annotation of the RNA-seq data

To identify key regulatory genes involved in the interspecific differences in reproductive organ organogenesis and development between the two *Liriodendron* species, the stamen primordia (LCS: Fig. 1A1–B1; LTS: Fig. 1A2-B2) and the gynoecium primordia (LCG: Fig. 2A1-B1; LTG: Fig. 2A2-B2) of *L. chinense* and *L. tulipifera* were sequenced by the high-throughput Illumina NovaSeq 6000 platform. Twelve cDNA libraries and a total of 124.70 G of raw reads were obtained. After low-quality reads were filtered out, 120.68 G of clean reads was selected for further analyses, and the Q30 was above 94.24% (Table 1). The RNA-seq reads were mapped onto the *L. chinense* reference genome using TopHat software, with 82.59-95.05% efficiency for each sample. The mapping rates of *L. chinense* were higher than those of *L. tulipifera*. Then, the mapped reads was spliced by Stringtie and Cufflinks software and compared with the genome annotation information to find the un-annotated transcriptional region and new transcripts or genes to supplement the genome annotation information. The mapped new transcripts were classified into nine groups according to the relationship of mapped transcripts and known transcripts (Supplementary Fig. S1). The x, i, j, u, and o groups represented the potential new transcripts, and the genes of the u group were named as new genes. In total, 42,268 genes were identified, including 6,999 new genes (named MSTRG.00000) and 35,269 reference genes (named Lchi00000 according to the genome data) (Supplementary Table S1). The reference genome was from *L. chinense*, while the RNA-seq samples came from *L. chinense* and *L. tulipifera*; therefore, we identified quite a large number of new genes that represented a good supplement to the *L. chinense* genome. The *L. chinense* reference genome consists of Scaffold0 to Scaffold3710, and the RNA-seq data were mapped onto 231 scaffolds. The number of reference genes mapped onto Scaffold723 was the highest among the scaffolds.

**Table 1:** Throughput, quality, and mapping rate of RNA-seq data.

Sample ID	Sample description	Clean reads	Q30%	Mapped reads
LCS	Stamen primordia of *L. chinense*	68,227,818	94.72	64,848,086 (95.05%)
		69,714,336	94.87	66,128,427 (94.86%)
		60,261,662	94.71	56,787,418 (94.23%)
LCG	Gynoecium primordia of *L. chinense*	63,363,042	94.51	59,404,859 (93.75%)
		78,243,242	94.86	74,177,083 (94.8%)
		73,722,554	94.92	69,606,598 (94.42%)
LTS	Stamen primordia of *L. tulipifera*	65,915,280	94.70	55,452,913 (84.13%)
		67,196,512	94.34	55,972,402 (83.3%)
		66,943,822	94.59	55,505,787 (82.91%)
LTG	Gynoecium primordia of *L. tulipifera*	68,457,226	94.24	56,692,095 (82.81%)
		70,174,788	94.50	58,514,132 (83.38%)
		63,953,200	94.74	52,821,472 (82.59%)

**Figure 3:**
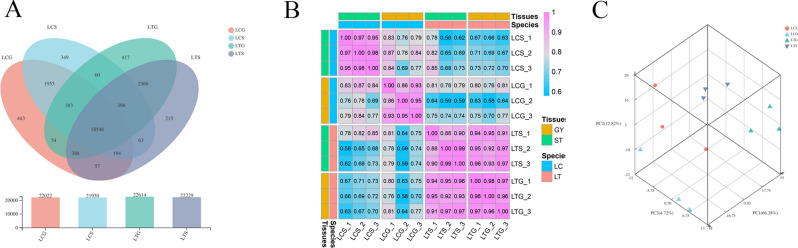
Sample relationship analysis based on all expressed genes. (**A**) Venn diagram among all samples; (**B**) Correlation between two samples analysis; (**C**) Principal component analysis.

In total, 36,769 genes were expressed in the four organs, with 21,950, 22,022, 22,614, and 22,229, in the LCS, LCG, LTS, and LTG, respectively, according to the criterion of TPM value ≥ 1 (Fig. 3A). The replicate samples were strongly correlated according to Pearson’s correlation coefficient (R^2^) (P < 0.01) (Fig. 3B). We also analyzed the principal components of the 12 samples, and they were clustered into four groups: LCS, LCG, LTS, and LTG (Fig. 3C). These results demonstrated that the four groups were distincted, and the differences among different groups were suitable for subsequent analyses.

### DEGs identified in the stamen primordia and gynoecium primordia of *Liriodendron* species

**Figure 4:**
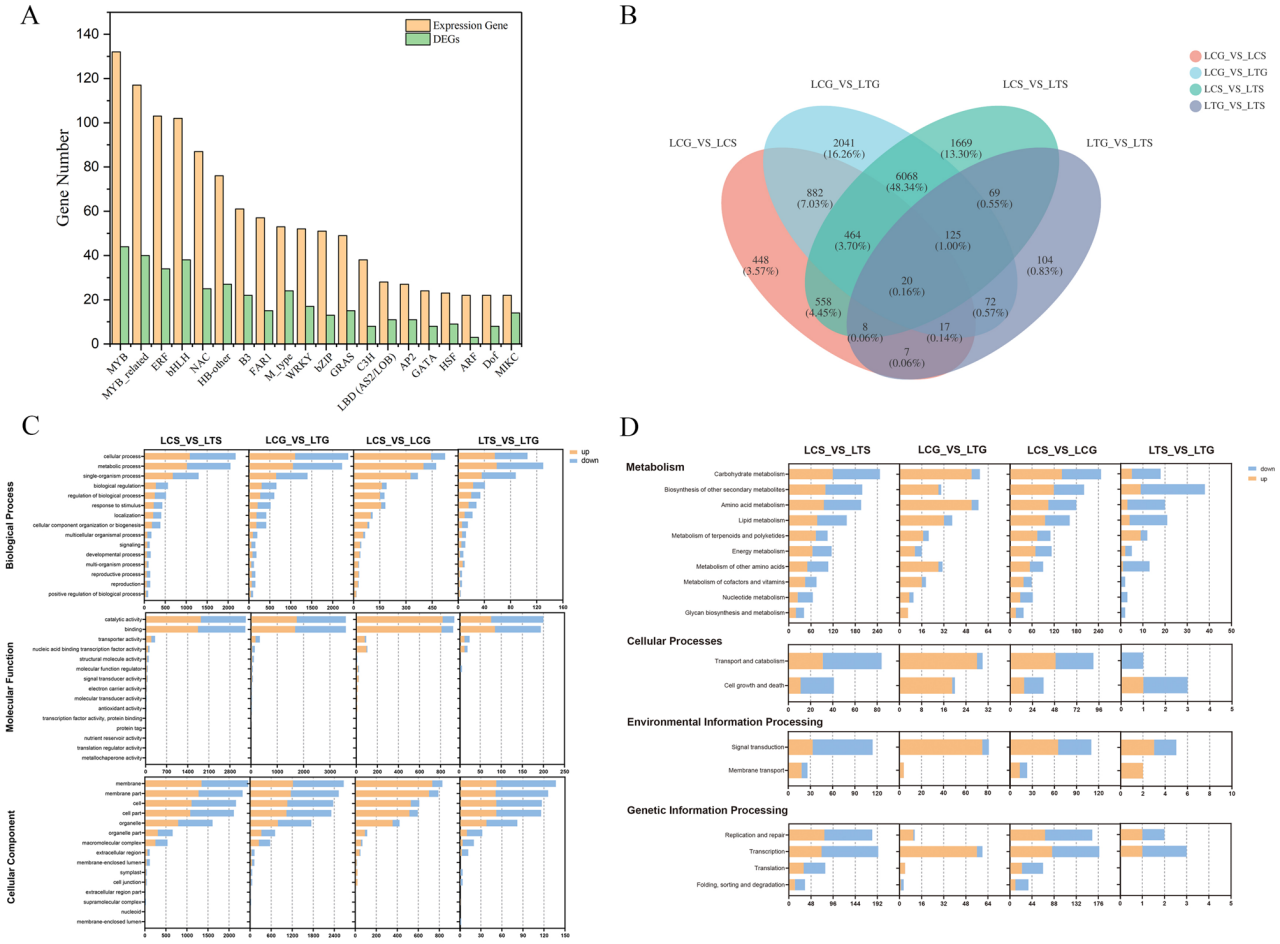
TF family prediction and GO functional annotation and KEGG pathway category analysis of DEGs. (**A**) TF family prediction of all expressed genes and all DEGs; (**B**) Number of DEGs identified in four comparisons; (**C**) GO functional annotation analysis of DEGs identified in four comparisons; (**D**) KEGG pathway category analysis of DEGs identified in four comparisons. Note: Fig. 4A was analyzed by Origin software (2017, https://www.originlab.com/).

According to the criteria of P-adjust < 0.05 and |log2FC|≥ 1, a total of 11,246 DEGs were identified among the four comparisons (LCS_vs_LCG, LCG_vs_LTG, LCS_vs_LTS, and LTS_vs_LTG). Moreover, 47 TF families were predicted by analyzing all expressed genes and 42 TF families were predicted for all DEGs, and they mainly included the MYB (44 genes), MYB-related (40 genes), bHLH (38 genes), ERF (34 genes), HB-other (27 genes), NAC (25 genes), and M-type (24 genes) families (Fig. 4A). The MYB, MADS-box, and bHLH families may be involved in the stamen and gynoecium organogenesis and development. Next, we performed an enrichment analysis of the DEGs identified in the four pairs of comparisons, i.e., two organ-specific and two species-specific comparisons. Based on the organ-specific comparison, we obtained 2,404 and 422 DEGs in *L. chinense* (LCS_vs_LCG) and *L. tulipifera* (LTS_vs_LTG), respectively. When species-specific comparisons were conducted, there were 8,981 and 9,689 DEGs in the stamen primordia (LCS_vs_LTS) and gynoecium primordia (LCG_vs_LTG), respectively (Fig. 4B; Supplementary Table S2).

The upregulated and downregulated DEGs of the four comparison pairs were annotated. The Gene Ontology (GO) terms were divided into three categories: molecular function (MF), cell component (CC), and biological process (BP) and the first categories of KEGG pathways were divided into metabolism, genetic information processing, cellular processes, environmental information processing, and organismal systems. In the GO functional annotation, a total of 52 GO terms were annotated. Relatively large numbers of genes were annotated to catalytic activity, binding (MF), membrane, membrane part, cell, cell part, organelle, organelle part (CC), cellular process, metabolic process, and single-organism process (BP) (Fig. 4C). The number of upregulated genes with GO functional annotations in LCS_vs_LCG was significant greater than that the number of downregulated genes. In the KEGG functional annotations, the number of DEGs assigned to carbohydrate metabolism, biosynthesis of other secondary metabolites, amino acid metabolism, lipid metabolism, folding, sorting and degradation, translation, signal transduction, and environmental adaptation was more than that in the other secondary categories of the KEGG pathways (Fig. 4D). The number of upregulated genes in LCG_vs_LTG was significant greater than that of downregulated genes, indicating that the genes identified from the gynoecium of *L. tulipifera* probably involved in more KEGG pathways. These results indicated that abundant metabolic activities and signal transduction occurred in the cells and the early stages of reproductive organs are important for the entire growth process of *Liriodendron* species.

**Figure 5:**
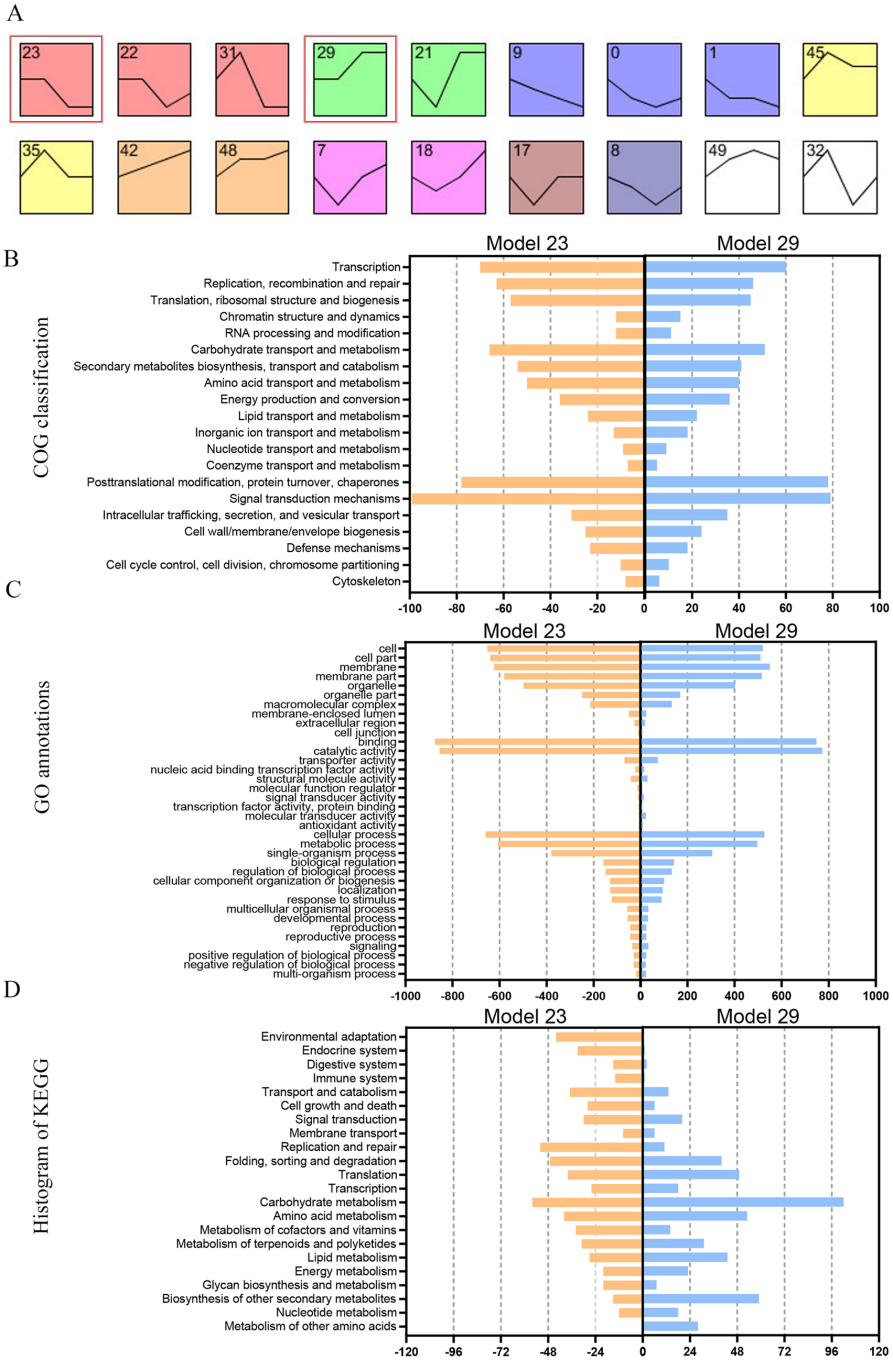
Expression clustering analysis between *L. chinense* and *L. tulipifera*. (**A**) Expression clustering of all samples; (**B**) COG classification analysis of Models 23 and 29; (**C**) GO functional annotation analysis of Models 23 and 29; (**D**) KEGG pathway category analysis of Models 23 and 29. (**A**) was conducted using STEM software (v1.3.8, http://www.cs.cmu.edu/~jernst/stem/) according to the criterion TPM ≥ 0.5 by the STEM clustering method and 16 model profiles were significant (P < 0.01).

To further explore the interspecific difference of reproductive organ organogenesis and development of *Liriodendron* species, we conducted a gene expression clustering analysis using Short Time-series Expression Miner (STEM) software. In total, 50 model profiles were obtained according to the criterion of TPM ≥ 0.5 by the STEM clustering method and 16 model profiles were significant (P < 0.01) (Fig. 5A). Model 23 (2,303 genes) and Model 29 (1,968 genes) showed obviously different gene expression levels between *L. chinense* and *L. tulipifera*; thus, the two models were used for functional annotations, including COG, GO and KEGG annotations (Fig. 5B–D). In Model 23 and Model 29, a similar number of genes was annotated by COG and GO terms, while the number differed greatly for the annotated KEGG pathways. In addition, the number of genes associated with cell growth and death (28 genes) and transport and catabolism (37 genes) identified by Model 23 was also higher than that by Model 29 (six genes and 13 genes, respectively). A high number of genes were annotated in carbohydrate metabolism (102 genes) and biosynthesis of other secondary metabolites (59 genes) in Model 29. We also conducted the KEGG enrichment analysis of Model 23 and Model 29 (P < 0.01), and the results revealed significant enrichment of five pathways in Model 23, namely, RNA polymerase, protein export, histidine metabolism, purine metabolism and propanoate metabolism; and significant enrichment of three pathways in Model 29, namely, monoterpenoid biosynthesis, starch and sucrose metabolism, cyanoamino acid metabolism. Interestingly, the monoterpenoid biosynthesis pathway was rather unique to *L. tulipifera*. The monoterpenoid biosynthesis may be related to the volatile composition and content of gynoecia, which influences pollination in plants. The seed-setting rate is higher in *L. tulipifera* than *L. chinense* in nature, and the results indicate that monoterpenoid biosynthesis may attract more insects, thereby improving the pollination of *L. tulipifera*.

### MADS-box family genes related to stamen and gynoecium development of *Liriodendron* species

We chose the MADS-box family and analyzed its expression patterns and functions during stamen and gynoecium development to explore the interspecific differences between the two *Liriodendron* species. A total of 44 MADS-box genes were identified from the *L. chinense* genome via Pfam annotation. Based on sequence analysis, 12 candidate MADS-box genes containing incomplete MADS-box domains were excluded from subsequent analyses. Therefore, 32 MADS-box genes were identified in the *Liriodendron* RNA-seq data and genome (Table 2). Among these genes, six genes were new genes and 26 genes were reference genes. These MADS-box genes encoded proteins with lengths from 136 to 476 amino acids, predicted molecular masses from 15.49 kDa to 53.49 kDa, and protein isoelectric points from 4.59 to 9.97.

**Table 2:** Identification of MADS-box family genes in the RNA-seq data.

Gene ID	ORF/bp	Chr. no	No. of introns	Type	Subfamily
Lchi01744	411	Scaffold1191	4	MIKC-type	PI-like
Lchi23168	633	Scaffold1505	6	MIKC-type	PI-like
MSTRG.22061	813	–	–	MIKC-type	AP3-like
Lchi02285	477	Scaffold682	3	MIKC-type	SVP-like
MSTRG.23969	697	–	–	MIKC-type	SVP-like
Lchi01587	669	Scaffold1191	6	MIKC-type	STK-like
Lchi04024	1143	Scaffold291	6	MIKC-type	AG-like
Lchi20361	654	Scaffold72	6	MIKC-type	SEP3-like
Lchi14062	1038	Scaffold1147	9	MIKC-type	B-sister-like
Lchi16005	516	Scaffold165	4	MIKC-type	B-sister-like
MSTRG.10046	597	–	–	MIKC-type	AGL12
MSTRG.18151	615	–	–	MIKC-type	AGL12
Lchi30451	900	Scaffold652	1	M-type	Mα
Lchi16146	672	Scaffold369	1	M-type	Mα
Lchi16145	744	Scaffold369	1	M-type	Mα
Lchi16144	1422	Scaffold369	1	M-type	Mα
MSTRG.1403	618	–	–	M-type	Mα
Lchi12579	1323	Scaffold1134	3	M-type	Mβ
Lchi32938	795	Scaffold1161	1	M-type	Mβ
Lchi12571	1098	Scaffold1134	1	M-type	Mβ
Lchi12568	651	Scaffold1134	2	M-type	Mβ
Lchi12576	1011	Scaffold1134	4	M-type	Mβ
Lchi34069	906	Scaffold1368	5	M-type	Mβ
Lchi12575	1431	Scaffold1134	5	M-type	Mβ
Lchi12578	1200	Scaffold1134	6	M-type	Mβ
Lchi12573	993	Scaffold1134	3	M-type	Mβ
MSTRG.20916	453	–	–	M-type	Mβ
Lchi25810	807	Scaffold1284	0	M-type	Mβ
Lchi00045	600	Scaffold915	1	M-type	Mγ
Lchi16590	1242	Scaffold2432	1	M-type	Mγ
Lchi01302	552	Scaffold432	8	M-type	Mδ
Lchi14336	933	Scaffold805	9	M-type	Mδ

**Figure 6:**
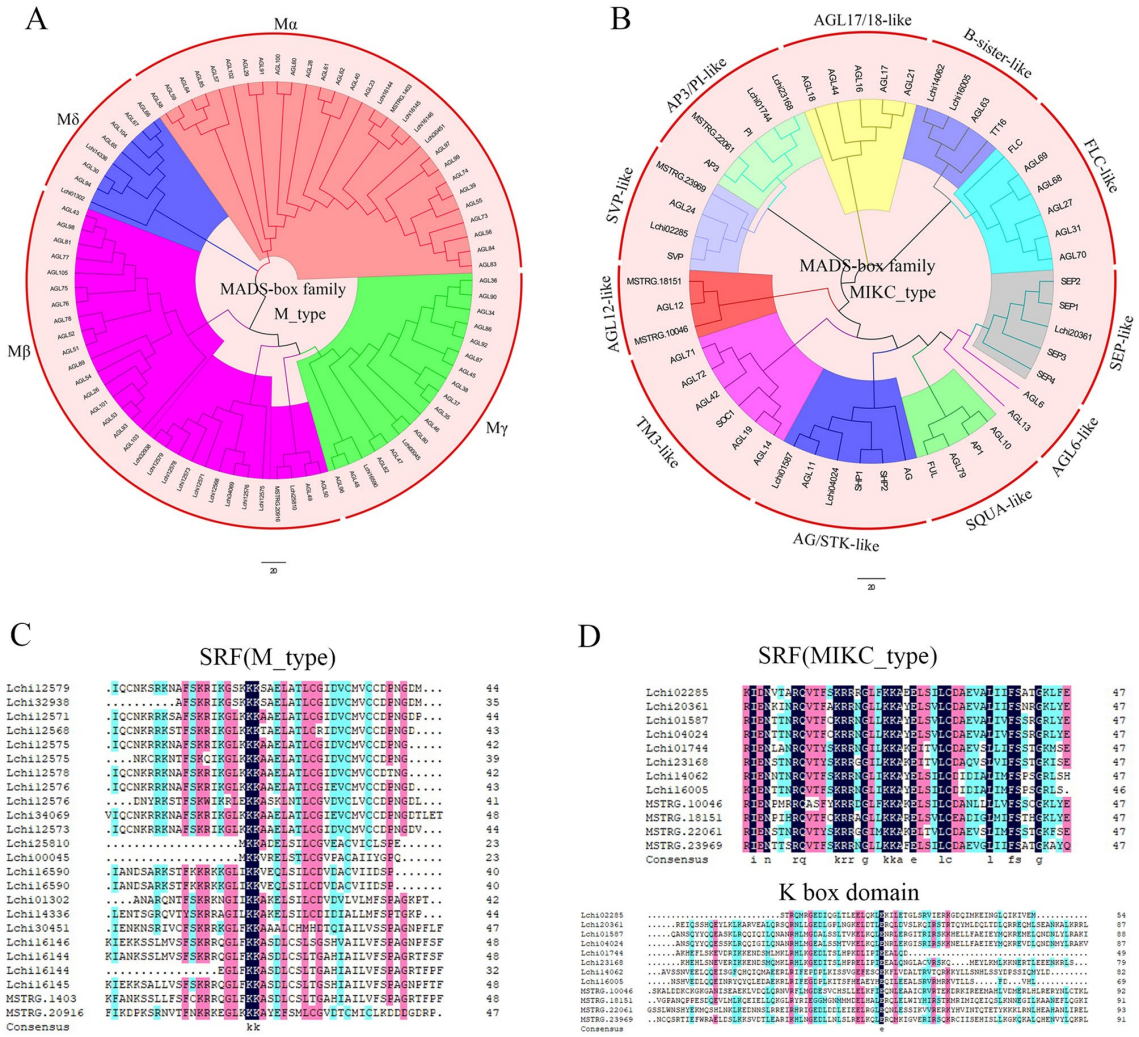
Construction of phylogenetic trees of the MADS-box proteins from *L. chinense* and *A. thaliana* and alignment of the SRF and K-box conserved domains. (**A**) Phylogenetic tree of type-I (M-type) MADS-box proteins from *L. chinense* (12) and *A. thaliana* (65) classified into 4 subgroups; (**B**) Phylogenetic tree of type-II (MIKC-type) MADS-box proteins from *L. chinense* (20) and *A. thaliana* (62) classified into 11 subgroups; (**C**) SRF conserved domain of M-type proteins; (**D**) SRF and K-box conserved domains of MIKC-type proteins. Notes: Phylogenetic trees were constructed by Clustal X (v2.1, http://www.clustal.org/clustal2/) and MEGA 7 software (https://www.megasoftware.net/) with the neighbor-joining (NJ) method with a bootstrap value of 1,000 and with the ML method, and they were edited using FigTree software (v1.4.3, http://tree.bio.ed.ac.uk/software/figtree/). The conserved domains of SRF and K-box were aligned using DNAMAN software (v10, https://www.lynnon.com/dnaman.html).

Phylogenetic trees were constructed based on the full-length sequences of MADS-box proteins from *L. chinense* and *A. thaliana* using the maximum likelihood (ML) method (Fig. 6A,B). The 32 MADS-box genes of *L. chinense* were classified into two types: M-type (20 genes) and MIKC-type (12 genes). Phylogenetic trees of both types were constructed using MADS-box protein sequences from both species. M-type MADS-box genes of *L. chinense* were further divided into four subgroups: Mα (five genes), Mβ (11 genes), Mγ (two genes), and Mδ (two genes) (Table 2). Among the MIKC-type MADS-box genes of *L. chinense*, we identified 12 MIKC-type genes and further divided them into 11 subgroups, namely, *AP3/PI-like*, *AGL17/18-like*, *B-sister*, *FLC-like*, *SEP-like*, *AGL6-like*, *SQUA-like*, *AG/STK-like*, *TM3-like*, *AGL12-like*, and *SVP-like*, according to the known groups of *A. thaliana* MADS-box genes. The *AP3/PI-like* subgroup consisted of B-class genes and contained three genes from *L. chinense*. Lchi01744 and Lchi23168 belonged to the *PI-like* class, and MSTRG.22061 belonged to the *AP3-like* class. The numbers of genes in the *SVP-like*, *AGL12*, *B-sister* and *AG/STK-like* subgroups were the same at two genes. The *SEP-like* subgroup consisted of E-class genes and included only one gene, Lchi20361. The *AG/STK-like* subgroup contained C/D class genes and included Lchi01587 and Lchi04024. Five subgroups, including *AGL17/18-like*, *FLC-like*, *AGL6-like*, *SQUA-like*, and *TM3-like,* were absent in the *L. chinense* genome. Subsequently, we analyzed the conserved domains of MADS-box proteins by alignment of their sequences (Fig. 6C,D). Both M-type and MIKC-type proteins had SRF domains, and the SRF domain was more conserved in the MIKC-type proteins than in the M-type proteins. In addition to the highly conserved SRF domain, the MIKC-type proteins also had a K-box domain. The K-box domain was obviously less conserved. A protein sequence alignment analysis of the SRF domain in *L. chinense* showed that the SRF domain was more conserved in MIKC-type proteins than that in M-type proteins. The domain induces the strongly conserved function and structure of MIKC-type proteins, which is consistent with previous study results.

**Figure 7:**
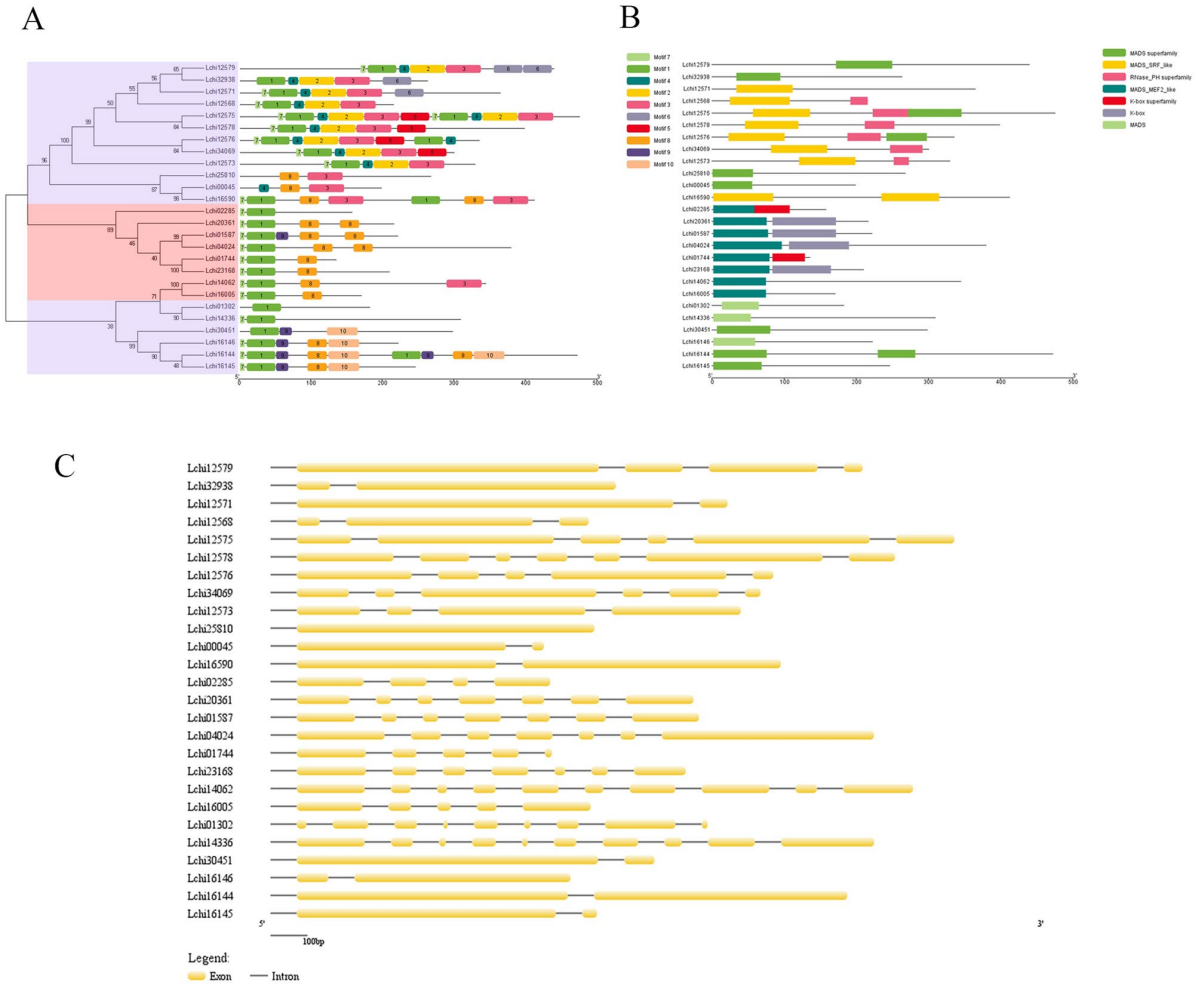
Distribution of conserved motifs (**A**), domains (**B**) and gene structures (**C**) of MADS-box proteins in *L. chinense*. All figures were analyzed by TB Tools software (v1.068, http://www.tbtools.com/).

The distributions of conserved motifs, domains, and introns of the MADS-box of *L. chinense* reference genes (26 genes) were analyzed by comparing the full-length cDNA and genomic DNA sequences of the genes. The conserved motifs and domains of MADS-box proteins in *L. chinense* were analyzed using MEME online software and TB tools. There were ten conserved motifs, named motifs 1–10 (Fig. 7A). Motif 1 represented the typical MADS domain and was found in all MADS-box proteins of *L. chinense* except Lchi25810 and Lchi00045. N-terminal motif 7 was found in most M-type and in all MIKC-type proteins. Motif 2 and motif 3 were specific to the Mβ subgroup, while motif 10 was specific to the Mα subgroup. The K-box domain was typical of MIKC-type proteins and absent in all M-type proteins, which was consistent with the results of conserved domain sequence alignments.

According to the results of the phylogenetic tree and conserved domain analyses, the Mδ-subgroup genes were more similar than the Mα-, Mβ-, and Mγ-subgroup genes to MIKC-type genes. Therefore, in several studies, the Mδ-subgroup genes were classified as MIKC-type genes. The 26 MADS-box reference genes of *L. chinense* mapped to the 17 scaffolds of the *L. chinense* genome (Fig. 7B; Table 2). Eight MIKC-type genes were randomly distributed on seven scaffolds. Scaffold1134 contained the maximum number of genes (seven genes), all of which belonged to the Mβ subgroup. All the genes in the Mα subgroup mapped to Scaffold369. These results indicated that the distribution of MIKC-type genes was more random than that of M-type genes. The structure of M-type genes significantly differs from that of MIKC-type genes. In general, M-type genes have only one or no introns, while MIKC-type genes have a large number of introns (five to eight introns). All MIKC-type MADS-box genes from *L. chinense* contained at least three and up to nine introns. In M-type MADS-box genes, the numbers of introns in the Mβ and Mδ subgroups were greater than those in the Mα and Mγ subgroups, while Lchi25810 was intronless (Fig. 7C; Table 2). Notably, the Mβ-subgroup genes had more introns (one to six) than the M-type genes. This unusual phenomenon in *L. chinense* cannot be explained at present. Interestingly, the Mδ-subgroup genes contained eight or nine introns, and their distribution was similar to that of MIKC-type genes, while the Mα- and Mγ-subgroup genes had no or only one intron. In *Arabidopsis*, Mδ genes can also be designated MIKC-type MADS-box genes based on their close relationships. In *L. chinense*, Mδ genes can also be MIKC-type genes according to their intron distribution. Our results support this classification.

### Expression patterns of MADS-box genes in different tissues of *Liriodendron* species

**Figure 8:**
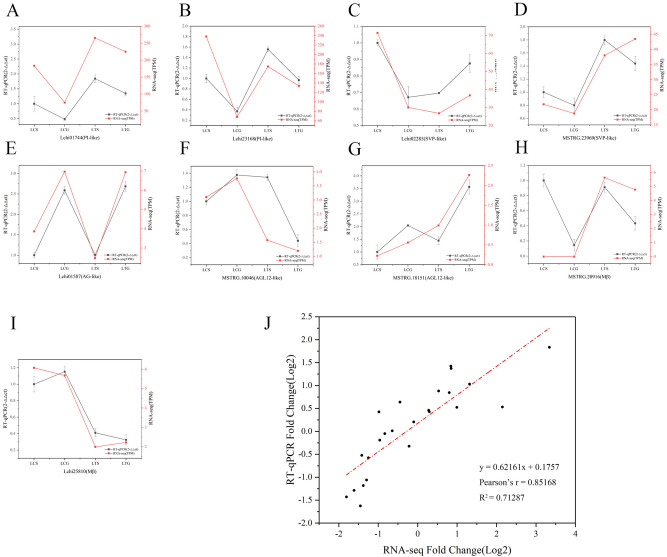
Validation of RNA-seq data by RT-qPCR assay. (**A–I**) Expression profiling of 9 MADS-box family genes; (**J**) Scatter plot showing the correlation between RNA-seq and RT-qPCR data. Note: All data and figures were analyzed by Origin software (2017, https://www.originlab.com/).

To calculate the accuracy of the RNA-seq data, we chose nine MADS-box genes for analysis by RT-qPCR, including seven MIKC-type genes, namely, Lchi01744, Lchi23168, Lchi02285, MSTRG23969, Lchi01587, Lchi25810, MSTRG10046, and MSTRG 18,151, and two M-type genes, namely, MSTRG20916 and Lchi28510. The TPM values were obtained for RNA-seq data. Then, we used the relative expression and TPM values to analyze the expression profiles with Origin software. The expression profiles of nine MADS-box genes were consistent between the RNA-seq and RT-qPCR data (Fig. 8). We calculated the log2-fold change (FC) values between the RNA-seq and RT-qPCR datasets to determine the correlation between them and analyzed the Pearson correlation coefficient. The results indicated that the RNA-seq and RT-qPCR datasets had a positive correlation coefficient (R^2^ = 0.71287) (Fig. 8J).

**Figure 9:**
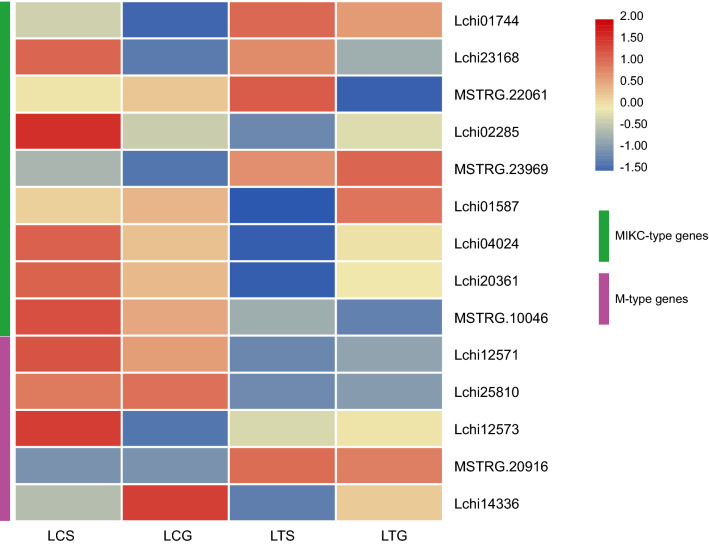
Heat map of highly expressed MADS-box genes in the stamen primordia and gynoecium primordia of *L. chinense* and *L. tulipifera*. Note: The heat map was analyzed using TB Tools software (v1.068, http://www.tbtools.com/).

The expression levels of the 32 MADS-box genes of *L. chinense* were obtained from the RNA-seq data. A total of 14 genes were chosen for heat map analysis by screening out the genes with low expression (average TPM value ≤ 1) in four tissues, including the stamen primordia and gynoecium primordia, of *L. chinense* and *L. tulipifera*. The 14 genes were expressed in at least one tissue, while the other 18 genes showed no or very low expression. Of the 18 genes with no or low expression, 15 genes were of M-type, and 3 genes were of MIKC-type. Among the 14 expressed genes, nine were MIKC-type genes (*AP3/PI*, *SVP*, *AG*, *SEP*, and *AGL12*), and the other five were M-type genes (Mβ and Mδ). Heat maps of 14 genes were constructed according to the expression levels of the genes in RNA-seq data after statistical normalization (Fig. 9). No M-type genes in the Mα and Mγ subgroups were expressed in the four tissues, and five genes in the Mβ (four genes) and Mδ (one gene) subgroups were expressed. All genes in the Mα and Mγ subgroups had no or low expression. The expression patterns in the stamen primordia and gynoecium primordia were similar in *L. chinense* and *L. tulipifera*. The interspecific difference in expression pattern was more obvious than the difference in expression pattern among tissues in the same species. Therefore, the MADS-box family is suitable for the interspecific difference analysis.

The new gene MSTRG.20916 (Mβ) was more highly expressed in *L. tulipifera* than in *L. chinense*, while the expression of Lchi12571 (Mβ) and Lchi25810 (Mβ) was lower in *L. tulipifera*. Conversely, for MIKC-type genes, the expression patterns in the same tissue were more similar than those in the same species. Lchi01744 (*PI-like*), Lchi23168 (*PI-like*), MSTRG.22061 (*AP3-like*), and Lchi02285 (*SVP-like*) had higher expression levels in the stamen primordia than in the gynoecium primordia. Lchi01587 (*STK-like*), Lchi04024 (*AG-like*), and Lchi20361 (*SEP3-like*) were expressed at lower levels in the stamen primordia than in the gynoecium primordia. The expression levels of MSTRG.23969 (*SVP-like*) in the stamen primordia and gynoecium primordia were similar in the same species, which was also observed for MSTRG.10046 (*AGL12-like*). These results showed that the new gene MSTRG.20916 may regulate mainly reproductive organ development of *L. tulipifera*. In addition, Lchi01744 (*PI-like*), Lchi23168 (*PI-like*), MSTRG.22061 (*AP3-like*), and Lchi02285 (*SVP-like*) may be involved in the stamen primordium development in the two *Liriodendron* species.

**Figure 10:**
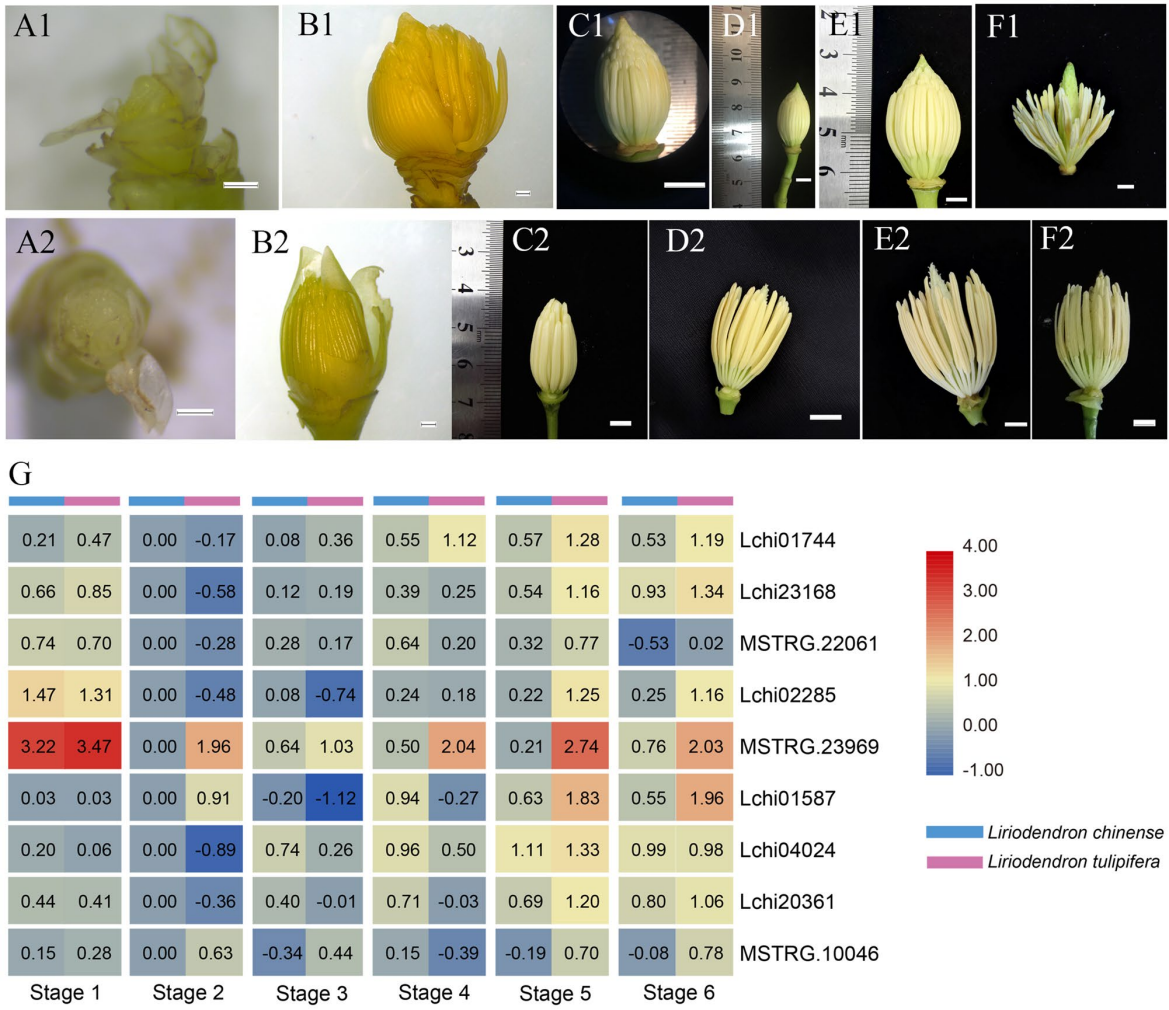
Heat map of the MIKC-type MADS-box genes in different stages of the stamens of *L. chinense* and *L. tulipifera*. (**A1–F1**) Stages 1–6 of the stamens of *L. chinense*; (**A2–F2**) Stages 1–6 of the stamens of *L. tulipifera*; (**G**) Heat map of the MIKC-type MADS-box genes of the stamens; (**A1**,**B1**,**A2**,**B2**) bar, 1 mm; C1-F1, C2-F2: bar, 5 mm. (**G**) was drawn using TB Tools software (v1.068, http://www.tbtools.com/).

**Figure 11:**
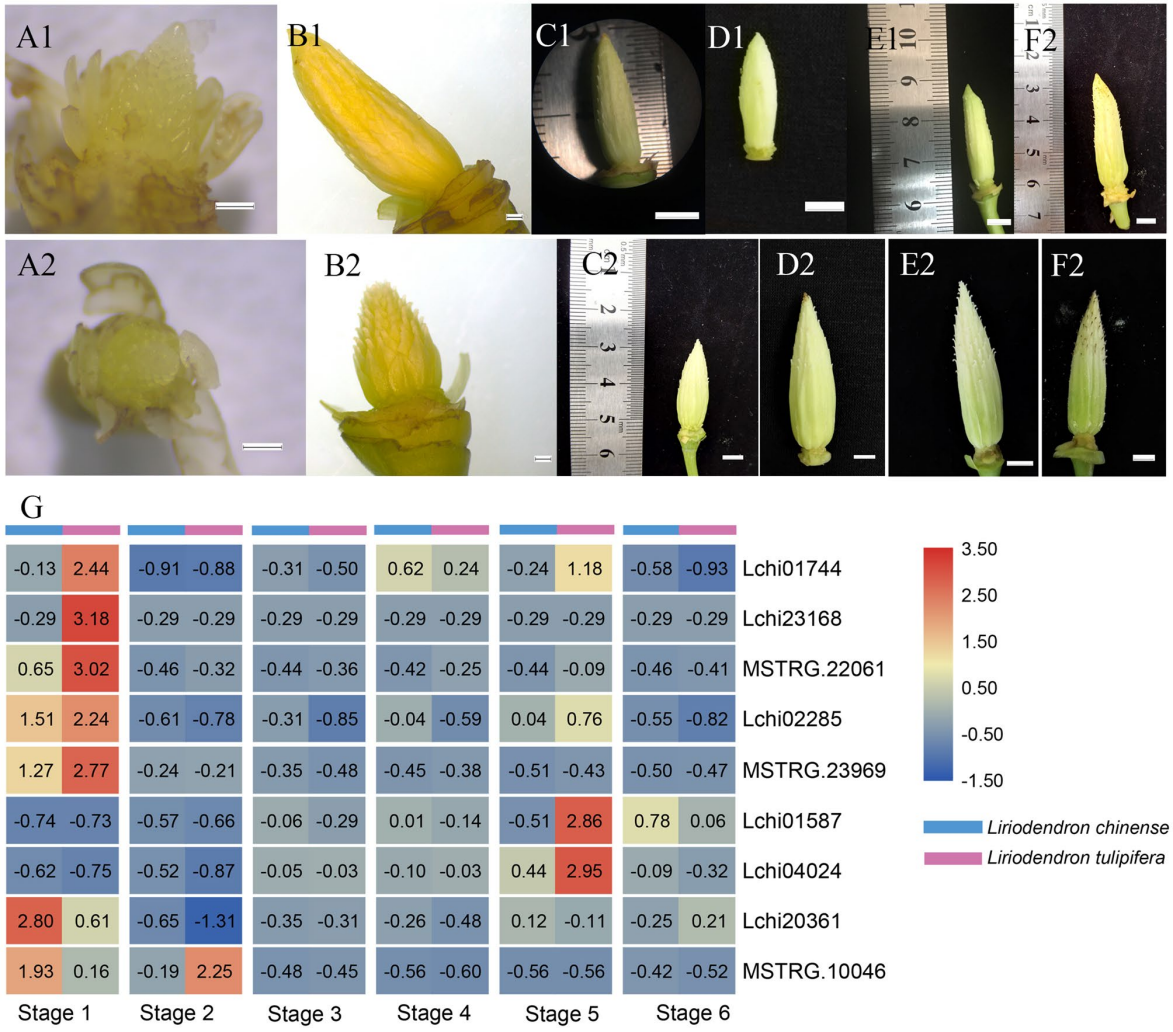
Heat map of the MIKC-type MADS-box genes in different stages of the gynoecia of *L. chinense* and *L. tulipifera*. (**A1–F1**) Stage 1–6 of the gynoecia of *L. chinense*; (**A2–F2**) Stage 1–6 of the gynoecia of *L. tulipifera*; (**G**) Heat map of the MIKC-type MADS-box genes of the gynoecia; (**A1,B1,A2,B2**) bar, 1 mm; (**C1-F1**,**C2-F2**) bar, 5 mm. Note: Fig. 11G was analyzed using TB Tools software (v1.068, http://www.tbtools.com/).

MADS-box genes were reported to have vital functions in floral organ identification, growth, and development, particularly MIKC-type genes. Therefore, we examined the species-specific expression patterns of nine highly expressed MIKC-type MADS-box genes by RT-qPCR assays after statistical normalization to further investigate their roles in interspecific differences of reproductive organ development. There were six stages of stamens (Fig. 10A–F) and gynoecia (Fig. 11A–F) in *L. chinense* and *L. tulipifera* from reproductive organ appearance to flower blossoming. The four reproductive organs of stage 1 (LCS, LTS, LCG, and LTG; Figs. 1A1, 1A2, 2A1, and 2A2) were also used as samples for RNA-seq. The organs of stage 2, stage 3 and stage 4 grew considerably after dormancy. Stages 4–6 were at anthesis and growth reached the peak in these genes. To visualize relatively small differences, the RT-qPCR data were normalized (log10 function) within the developmental stage for each gene.

The nine MIKC-type genes were all expressed in the 12 samples of stamens (Fig. 10G). Two *SVP-like* genes, namely, Lchi02285 and MSTRG.23969 were highly expressed at the stamen primordia stage (stage 1) of the two species, especially MSTRG.23969 (*SVP-like*). In addition, at other stages, the expression level of MSTRG.23969 (*SVP-like*) in *L. tulipifera* was much higher than that in *L. chinense*. The expression of most genes declined at stage 2 and stage 3. During stage 4 to stage 6, the expression of all genes obviously increased except that of MSTRG.22061 (*AP3-like*). As shown on the heatmap, the expression level in *L. tulipifera* was consistently higher than that in *L. chinense*. Thus, MSTRG.23969 (*SVP-like*) may be involved in the early stage, and *PI-like*, *AG/STK-like* and *SEP3-like* may participate in the later stages of stamen development.

Significantly more genes presented differential expression in gynoecia than that in stamens, and nine MIKC-type genes showed high expression in gynoecia (Fig. 11G). Most genes displayed higher expression at stage 1 than other stages except for the two *AG/STK-like* genes. Moreover, six genes presented significantly higher expression in *L. tulipifera* than that in *L. chinense*. Lchi20361 belongs to the *SEP-like* subgroup (E-class) and showed higher expression in the gynoecium primordia of *L. chinense* than at other stages, while for *L. tulipifera*, the Lchi20361 gene presented higher expression at all stages. At the anthesis stage (stage 5) of gynoecium development, the expression levels of Lchi01744 (*PI-like*), Lchi01587 (*STK-like*) and Lchi04024 (*AG-like*) were much higher in *L. tulipifera* than in *L. chinense*. These results indicated that these B-class and *SVP-like* genes might regulate the gynoecium primordia development of *L. tulipifera*. Moreover, B-class, *AG/STK-like*, *AGL12-like* and *SVP-like* genes play important roles in reproductive organ formation and development.

## Discussion

Although one flower of *L. chinense* can produce one million pollen grains, the seed-setting rate is less than 10% under natural conditions and far less than that of *L. tulipifera*^8,35^. Over the last twenty years, many researchers have focused on investigating the reasons for the low seed-setting rate in *L. chinense*. Pollination, resources, and genetic loads are likely to limit the seed-setting rate^8^. Obviously, the seed-setting rate is closely related to stamen and gynoecium development. According to morphological and proteomic analyses, Li et al. suggested that a pistil feature might be the main reason for the low seed-setting rate in *L. chinense*^6^. Morphological and anatomical observations of the reproductive organs showed that *L. chinense* and *L. tulipifera* had similar organ shapes. Although *L. chinense* (59) had more stamens than *L. tulipifera* (38), the seed-setting rate was lower. The pollens of *Liriodendron* species are redundant, and larger number of pollens does not improve the pollination. Although *L. chinense* possesses a higher number of gynoecia than *L. tulipifera*, the frequency of forming two ovules in *L. tulipifera* is higher than that in *L. chinense*. Furthermore, *L. chinense* is vulnerable to abortive gynoecia. In addition, a number of DEGs related to monoterpenoid biosynthesis were enriched in this investigation. Monoterpenoids may be related to the volatile composition and content of gynoecia, which influences pollination in plants. Therefore, we speculate that the gynoecia may be the key element in *Liriodendron* that affects the seed-setting rate, and abortive gynoecia and monoterpenoid biosynthesis may lead to a great difference in the seed-setting rate between *L. chinense* and *L. tulipifera*.

Floral organ development and flowering time are important agronomic traits in breeding and production for directly determining the adaptability and commerciality of plants^36,37^. The MADS-box genes, particularly the plant-specific MIKC-type genes, play important roles in flower development. MIKC-type genes are well studied in angiosperms. Several recent studies characterized the MIKC genes in Magnoliaceae species, including *Magnolia wufengensis*, *M. grandiflora* and *M. sinostellata*, and identified 20 DEGs of the MADS-box and AP2 family in *Magnolia sinostellata*; these genes were expressed at high levels during flower bud differentiation^38,39^. In this study, we identified 32 MADS-box genes. The number of MADS-box genes in Magnoliaceae species is much lower than that in core angiosperms, such as *A. thaliana* (107 genes), *Oryza sativa* (75 genes), *Malus domestica* (145 genes), and *B. oleracea* (91 genes)^30,40^. We know that the size of the genome is disproportionate to the number of MADS-box genes and that different species have different numbers of such genes. The MADS-box is thought to have originated from several gene duplication events that led to neofunctionalization, subfunctionalization or nonfunctionalization^41^. During the evolution of flowering plants, the number of MADS-box families increased^18^. Considering the phylogenetic position of the basal angiosperm *Liriodendron* species, the MADS-box genes in these species may have undergone fewer duplication events than those in core angiosperms, which led to fewer members.

B-class (*AP3/PI-like*) genes play a major role in specifying stamen development^13^. In loss of B-class genes mutants, the stamens are transformed into carpels. *AP3/PI-like* proteins function by forming obligate heterodimers in eudicot species or higher complexes with A-, C-, or E-class proteins to regulate the development of petals or stamens, respectively^13,26^. Two *AP3* homologs, *MAwuAP3_1/2*, and one *MawuPI* were isolated from *M. wufengensis*, and they were restricted to the tepals and stamens. Three B-class genes (two *PI* and one *AP3*) were identified in *Liriodendron* species, and MSTRG.22061 (*AP3*) was newly identified in the *Liriodendron* genome. The two *PI* genes Lchi01744 and Lchi23168 had the highest expression among other MADS-box genes in the stamen primordia. In addition, they displayed high expression at anthesis and always showed higher expression in *L. tulipifera* than in *L. chinense*. The expression pattern was also found in C-, D-, E-class genes. The C/D class, including *AG*, *SHATTERPROOF* (*SHP1*)/*AGL1*, *SHP2*/*AGL5*, and *SEEDSTICK* (*STK*)/*AGL11*, is also mainly involved in the development of reproductive organs, fruit ripening and seed dispersal^31,42^. The *AG*/*STK* genes are restricted to core eudicots and grasses, and their functions are limited in basal angiosperms. Ma et al. identified three genes of the *AG/STK* subgroup (*MawuAG1*, *MawuAG2* and *MawuSTK*) in *M. wufengensis*. *AG*-lineage genes were expressed in both stamens and carpels, and the expression of *STK*-lineage genes in the stamens was lost soon after the appearance of the *STK* lineage^43^. Lchi04024 (*AG/STK*) presented a similar expression pattern as *PI-like* genes. Lchi01587 (*AG/STK*) was expressed differently in the stamens between *L. chinense* and *L. tulipifera* and only exhibited high expression at anthesis in *L. tulipifera*.

E-class (*SEP-like*) genes regulate floral meristem formation from vegetative organs and have obvious partially redundant functions during flower development^28,44,45^. Moreover, these genes do not function alone in flower development but rather form complexes with other classes of proteins^44,46^. The complexes are essential in plants; therefore, many researchers have suggested that the E-class floral homeotic proteins are more important than the other classes of proteins^47^. Roxana et al. pointed out that the *SEP-like* genes have undergone several duplication events by performing phylogenetic analyses and that the two copies of *SEP3* have evolved under balancing selection because of their critical roles in floral organ specification^48^. The first duplication occurred prior to the origin of extant angiosperms, resulting in the *AGL2/3/4* (*SEP1/2*) and *AGL9* (*SEP3*) clades, and the second duplication occurred within these clades of eudicots and monocots^49,50^. Moreover, *SEP-like* genes have been identified in all angiosperms, including several basal angiosperms, although they have not yet been detected in gymnosperms^49^. In this study, we identified only one *SEP-like* gene in the *Liriodendron* species: *SEP3* (Lchi20361). The *SEP-like* gene in *Liriodendron* species may have undergone the first duplication, resulting in the *SEP3* clades. The expression of Lchi20361 was second only to that of the *PI-like* genes in the stamen primordia, and they displayed similar expression pattern. The *PI-like* genes, Lchi04024 (*AG/STK*) and Lchi20361 (*SEP3*) may play an equally important role at early stamen development for the two *Liriodendron* species, and their expression model is conversed. During the maturation of stamens and pollens, *PI-like*, Lchi01587 (*AG/STK*) and Lchi20361 (*SEP3*) are vital for *L. tulipifera*.

Lchi20361 (*SEP3*) presented the highest expression among the MADS-box genes in the gynoecium primordia, and its expression in other stages declined sharply in the *Liriodendron* species. Two *PI-like* genes were second to the *SEP3* gene and were significantly expressed in the gynoecium primordia. In addition, the expression models of *SEP3-like* genes were similar to that of the *PI-like* genes. *SVP-like* genes are negative regulators of floral meristem identity and involved in the regulation of the flowering transition and the integration of signals from different flowering pathways along with *FLC*, *FT*, *LFY*, and *SCO1-like* genes^51,52^. Three *SVP-like* homologous genes, *OsMADS22/47/55*, were expressed in the buds, roots and flowers^53^. Four *SVP-like* genes (*SVP1/2/3/4*) in kiwifruit were expressed in vegetative tissues^54^. Five *SVP-like* genes (*VvSVP1*/*2*/*3*/*4*/*5*) were identified in grapevine and found to be differentially expressed in the shoots, leaves, stems, flowers, and fruits, similar to the pattern in *Arabidopsis*^55^. Two *SHORT VEGETATIVE PHASE* (*SVP-like*) genes, namely, MSTRG23969 and Lchi02285, were identified in *Liriodendron* and found to be highly expressed in the gynoecium primordia, and their expression patterns were also similar to that of *PI-like* genes. Obviously, *SEP3* and *PI-like* genes function in early development of gynoecia. However, *SEP3* is more likely to regulate the process in *L. chinense*, and *PI-like* and *SVP-like* genes are more likely to regulate the process in *L. tulipifera*. The expression levels of two *AG/STK-like* genes increased sharply at stage 5 (anthesis) of gynoecia in *L. tulipifera*. Since the C/D-class genes are involved in the development of carpels and ovules, *AG/STK-like* genes may regulate the maturation process of carpels and ovules in *L. tulipifera*. Considering the significant interspecies difference of *AG/STK-like* genes between two *Liriodendron* species, we believe that further functional research on *AG/STK-like* genes can provide new insights into interspecific differences of carpels and ovules in *Liriodendron*.

Compared to information on the functions of MIKC-type MADS-box genes, information on M-type genes is very limited. Several studies in *Arabidopsis* indicated that M-type genes participate in plant growth and reproduction, particularly in female gametophyte, embryo, and endosperm development^40^. Although the M-type genes outnumber the MIKC-type genes, the function of the M-type genes in plants is not well understood. The difficulty in characterizing M-type genes lies in their low expression levels in plants. In this study, only five of 22 M-type genes were expressed in reproductive tissues (TPM ≥ 1) and the expression of one gene, Lchi14336 (Mδ), was relatively high. Mδ genes can also be considered MIKC-type genes based on their expression patterns and intron distributions^17^. The expression level of M-type genes was significantly lower than that of MIKC-type genes in the *Liriodendron* species, and these genes are rarely be involved in reproductive primordium development.

## Materials and methods

### Plant materials

The plant materials were collected from two sample trees of *L. chinense* (provenance: Jiangxi, China) and *L. tulipifera* (provenance: Georgia, USA) in a trial plantation of *Liriodendron* species located in Zhenjiang, Jiangsu Province (119° 13′ 20″ E, 32° 7′ 8″ N)^1,56^. The flower buds, stamen primordia, and gynoecium primordia were collected from June 2019 to October 2019 at intervals of one week, and the six stages of stamen and gynoecium were collected from February 2019 to June 2019 at intervals of 10–15 days. Some materials were stored at − 80 °C for RNA extraction, and the remaining material was fixed in formaldehyde-acetic acid-alcohol (FAA) for further use in scanning electron microscopy (SEM) and paraffin sectioning.

### Morphological and cytological observation

The stamen primordia and gynoecium primordia were dissected carefully and photographed using a LEICA S6D stereomicroscope (Leica Microsystems, Wetzlar, Germany). Then, the floral buds, stamen primordia, and gynoecium primordia were fixed in FAA for 24 h for SEM and paraffin sectioning. Some fixed materials were dehydrated in an ethanol series for 20 min per step and dried using CO_2_ as the exchange agent by an EMITECH K850 critical point dryer (Emitech, Canterbury, British). Then, the samples were coated with gold by an Edwards E-1010 ion sputter golden coater (Hitachi, Tokyo, Japan) and photographed with an FEI Quanta 200 scanning electron microscope (FEI, Eindhoven, Netherlands).

The remaining fixed materials were dehydrated in an ethanol series, transparentized in a xylene series, infiltrated in paraffin, embedded in paraffin wax, and sectioned at an 8 µm thickness with a LEICA RM2145 rotary microtome (Leica Microsystems, Wetzlar, Germany). Finally, the sections were stained with safranin and fast green and photographed with a Zeiss AXIO Axioscope A1 fluorescence microscope (Carl Zeiss, Jena, Germany).

### RNA extraction, illumina NovaSeq 6000 sequencing and read mapping

Total RNA was extracted from the reproductive tissue (stamen primordia and gynoecium primordia) of *L. chinense* and *L. tulipifera* using Plant RNA Purification Reagent according to the manufacturer’s instructions (Invitrogen, Waltham, USA). Then, the RNA quality was determined by a 2100 Bioanalyzer (Agilent, California, USA), and the RNA was quantified using an ND-2000 (NanoDrop Technologies, Wilmington, USA). Only high-quality RNA samples were used to construct a sequencing library. RNA purification, reverse transcription, library construction and sequencing were performed by Majorbio Biopharm Biotechnology (Shanghai, China) according to the manufacturer’s instructions (Illumina, San Diego, CA). The paired-end RNA-seq library was sequenced with an Illumina NovaSeq 6000 instrument (2 × 150 bp). The raw paired-end reads were trimmed and quality controlled by SeqPrep and Sickle with default parameters. Then, clean reads were separately aligned to the *L. chinense* genome (NCBI, CNA0002404) with orientation mode using TopHat software (v2.1.1, http://ccb.jhu.edu/software/tophat/index.shtml)^34,57^. The genes were annotated with the NR (ftp://ftp.ncbi.nlm.nih.gov/blast/db/), Swiss-Prot (http://www.gpmaw.com/html/swiss-prot.html), KEGG (https://www.genome.jp/kegg/), EuKaryotic Orthologous Groups (KOG, ftp://ftp.ncbi.nih.gov/pub/COG/KOG/), Pfam (http://pfam.xfam.org/), and GO (http://geneontology.org/) databases^58–63^.

### Differential expression analysis and functional enrichment

To identify differentially expressed genes between two different samples, the expression level of each transcript was calculated using the TPM method according to the following criteria: P-adjust < 0.05 and |log2FC|≥ 1. RSEM (v1.3.1, http://deweylab.github.io/RSEM/) was used to quantify gene abundance^64^. In addition, functional enrichment analyses, including GO and KEGG analyses, were performed to identify the DEGs that were significantly enriched in GO terms and metabolic pathways at a Bonferroni-corrected P-value ≤ 0.05 compared with the whole background transcriptome. GO functional enrichment and KEGG pathway analyses were carried out by Goatools (v1.0.11, https://pypi.org/project/goatools/) and KOBAS (v2.1.1, http://kobas.cbi.pku.edu.cn/kobas3)^65,66^. The gene expression clustering analysis was conducted using STEM software (v1.3.8, http://www.cs.cmu.edu/~jernst/stem/) according to the criterion TPM ≥ 0.5 by the STEM clustering method, and 16 model profiles were significant (P < 0.01)^67^.

### MADS-box family phylogenetic analysis and conserved domain sequence alignment

*Arabidopsis* MADS-box protein sequences were downloaded from the Plant Transcription Factor Database (v3.0, PlnTFDB, http://plntfdb.bio.uni-potsdam.de/v3.0/) and the Arabidopsis Information Resource (TAIR, https://www.arabidopsis.org/) and used for phylogenetic analysis^68,69^. The candidate genes with MADS domains (PF00319; PF01486) were chosen from the Pfam database (http://pfam.xfam.org/). The MADS-box protein sequences of *A. thaliana* and *L. chinense* were aligned with Clustal X (v2.1, http://www.clustal.org/clustal2/) with default parameters, and a phylogenetic tree was constructed in MEGA 7 software (https://www.megasoftware.net/) with the neighbor-joining (NJ) method with a bootstrap value of 1,000 and with the ML method^70,71^. Then, the phylogenetic tree was edited esthetically using FigTree software (v1.4.3, http://tree.bio.ed.ac.uk/software/figtree/). The conserved domains, including SRF (PF00319) and K-box (PF01486), of MADS-box proteins of *L. chinense* were aligned using DNAMAN software (v10, https://www.lynnon.com/dnaman.html) with default settings.

### Conserved motif and gene structure analyses

The conserved motifs of the MADS-box proteins in *L. chinense* were predicted by Multiple Em for Motif Elicitation online software (MEME, v5.1.1, http://meme-suite.org/tools/meme) and analyzed using TB Tools software (v1.068, http://www.tbtools.com/)^72,73^. The gene structures of MADS-box proteins in *L. chinense* were constructed by the Gene Structure Display Server online software (GSDS 2.0, http://gsds.cbi.pku.edu.cn/)^74^.

### Real-time quantitative PCR (RT-qPCR)

To verify the RNA-seq accuracy and research the expression profiles of the MADS-box genes in *L. chinense* and *L. tulipifera*, a total of 14 genes were analyzed by RT-qPCR using *eIF3* as a reference gene^75^. Total RNA was extracted using an RNAprep Pure Kit (Tiangen, China), and cDNA was synthesized by the PrimeScript RT enzyme with a gDNA eraser (Takara, Japan). Then, RT-qPCR was performed on an ABI StepOne Plus thermal cycler (Applied Biosystems, California, USA) using a TB Green SYBR Premix Ex Taq Kit (Takara, Shiga, Japan). All primers were designed by Oligo7 software (http://www.oligo.net/index.html) and are listed in Supplementary Table S3^76^. The relative expression profiles of 14 genes were analyzed using the 2^−ΔΔCT^ method, and the photographs and the correlations between RNA-seq and RT-qPCR data were analyzed and visualized by Origin software (2017, https://www.originlab.com/).

## Conclusions

In conclusion, we examined the stamen primordium and the gynoecium primordium of two *Liriodendron* species via scanning electron microscopy combined with paraffin sectioning. The size and number of stamens and gynoecia in *L. chinense* were greater than that those in *L. tulipifera.* The plentiful pollens may be redundant and are not the main causes of low seed-setting rate of *L. chinense*, more likely, the abortive gynoecia are related to the low seed-setting rate in *L. chinense*. A total of 12 libraries were constructed for the stamen primordium and gynoecium primordium from the two species in an RNA-seq assay, and 42,268 genes were identified, including 6,999 new genes and 35,269 reference genes, of which 34,216 (80.95%) genes were annotated. Monoterpenoid biosynthesis was enriched in *L. tulipifera*, and the monoterpenoid contenst may be related to the difference in seed setting rate between the two species. Then, we selected an important TF family, the MADS-box family, for further study. A total of 32 MADS-box genes were found to contain complete MADS-box domains, including six new genes and 26 reference genes. The expression analysis of nine highly expressed MIKC-type genes during six stages of stamen and gynoecium development indicated that the *PI-like*, *AG/STK-like*, *SEP-like*, and *SVP-like* genes may play important roles in the differentiation in organogenesis and development of reproductive organs between the two *Liriodendron* species.

## Supplementary Information

Supplementary Information
