# Supplementary material for: Genome-wide identification of MIKC-type genes related to stamen and gynoecium development in Liriodendron
Source: Sci Rep. 2021 Mar 22;11:6585. doi: 10.1038/s41598-021-85927-7 (PMC7985208; doi:10.1038/s41598-021-85927-7)
Supplement: Supplementary file 1 — Supplementary Information [file 41598_2021_85927_MOESM1_ESM.pdf]

Genome-wide identification of MIKC-type genes related to stamen and gynoecium development in *Liriodendron*

Huanhuan Liu[1], Lichun Yang[1], Zhonghua Tu[1], Shenghua Zhu[1], Chengge Zhang[1], Huogen Li[1*]

[1]Key Laboratory of Forest Genetics & Biotechnology of Ministry of Education, Co-Innovation Center for Sustainable Forestry in Southern China, Nanjing Forestry University, Nanjing 210037, Jiangsu, China

Supplementary Table S1 Mapped and annotated genes in the RNA-seq data

Type	Total number	No. of annotation
All transcript	68,979	59,052
New transcript	33,710	28,787
Reference transcript	35,269	30,265
Expression transcript	61,607	53,846
All gene	42,268	34,216
New gene	6,999	3,951
Reference gene	35,269	30,265
Expression gene	36,769	30,778

Supplementary Table S2 Functional annotations of RNA-seq data

	GO	KEGG	COG	NR	Swiss-Prot	Pfam	Total_anno	Total
Expression genes (%)	24228 (0.6589)	11782 (0.3204)	26371 (0.7172)	30422 (0.8274)	22946 (0.6241)	25379 (0.6902)	30778 (0.8371)	36,769 (1)
Expression transcripts (%)	43,610 (0.7079)	22,756 (0.3694)	48,040 (0.7798)	53,381 (0.8665)	42,142 (0.684)	44,494 (0.7222)	53846 (0.874)	61,607 (1)
Reference genes (%)	23,165 (0.6568)	11,348 (0.3218)	25,718 (0.7292)	29,847 (0.8463)	22,379 (0.6345)	25,977 (0.7365)	30265 (0.8581)	35,269 (1.0)
Reference transcripts (%)	23,165 (0.6568)	11,348 (0.3218)	25,718 (0.7292)	29,847 (0.8463)	22,379 (0.6345)	25,977 (0.7365)	30,265 (0.8581)	35,269 (1.0)
New genes (%)	3,097 (0.4425)	12,86 (0.1837)	3,080 (0.4401)	3,876 (0.5538)	2,424 (0.3463)	2,151 (0.3073)	3,951 (0.5645)	6,999 (1.0)
New transcripts (%)	23,944 (0.7103)	13,062 (0.3875)	26,391 (0.7829)	28,592 (0.8482)	23,021 (0.6829)	22,712 (0.6737)	28,787 (0.854)	33710 (1.0)
All genes (%)	26,262 (0.6213)	12,634 (0.2989)	28,798 (0.6813)	33,723 (0.7978)	24,803 (0.5868)	28,128 (0.6655)	34,216 (0.8095)	42,268 (1.0)
All transcripts (%)	47,109 (0.6829)	24,410 (0.3539)	52,109 (0.7554)	58,439 (0.8472)	45,400 (0.6582)	48,689 (0.7059)	59,052 (0.8561)	68,979 (1.0)

Supplementary Table 3 Numbers of DEGs in the four comparisons

	LCS_vs_LCG	LCG_vs_LTG	LCS_vs_LTS	LTS_vs_LTG
total	2,404	9,689	8981	422
down	304	5,172	4,281	237
up	2,100	4,517	4,700	185

Supplementary Table S4 The primer sequences of RT-qPCR

Primer names	Primer sequences
MSTRG.10046F	CGCAGTCCTTCCCATCTTAGTGT
MSTRG.10046R	CTCCCATCAAGAATCGCACGTT
MSTRG.20916F	GCGACGAAGTTCATCAAAGACCC
MSTRG.20916R	GTCTATCGCCGTCATCGTCCT
MSTRG.22061F	ATTGCAGCCCTTCTACAACGAC
MSTRG.22061R	ATCTTCACCGATCCTATGCCT
MSTRG.23969F	GCTCATCATCTTCTCCGCCACT
MSTRG.23969R	ATTCTTGCCTCCAACGTGTCC
Lchi20361F	TGCAACTACGGAGCACCAGA
Lchi20361R	CGTCTAGCTGCCTTTCAAGTGT
MSTRG.18151F	GGTCCTGCGAACCAACCTCC
MSTRG.18151R	ATCCGCATCTTTGTAGAGCGAA
Lchi02285F	TTCTCTGCCACCGGAAAGCTC
Lchi02285R	AGATGCACCCATTTGTGACTGA
Lchi04024F	TCGTCTTCTCCAGCCGTGGTC
Lchi04024R	ATCCCTATTTGTTGGCGCAGT
Lchi01587F	CCAACAGCAGCATAAAAGCAACA
Lchi01587R	CCCATTAAGTGCCGATTTGCAT
Lchi01744F	ATCTTCTCCAGCACCGGCAAG
Lchi01744R	TGATGTCAGATCCTCGCCCTT
Lchi25810F	CCGATGCCCTTTCCATGCTT
Lchi25810R	AGTGTAGAAATCCGCTCACGAAC
Lchi23168F	CCCTCGTCATCTTCTCTAGCAC
Lchi23168R	ATCCTCGCCCTTCAAATGCC

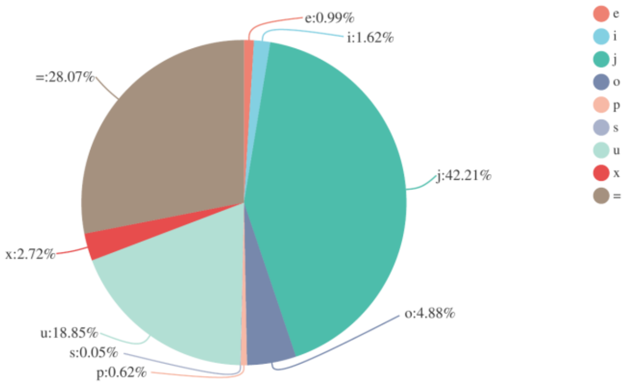

Supplementary Figure S1 Classification of new transcripts. =: Complete match of intron chain; e: Single exon transfrag overlapping a reference exon and at least 10 bp of a reference intron, indiction a possible pre-mRNA fragment; i: Transfrag falling entirely within a reference intron; j: Potentially novel isoform (fragment):at least one splice junction is shared with a reference transcript; o: Generic exonic overlap with a referfence transcript; p: Possible polymerase run-on fragment(within 2Kbases of a reference transcript; s: An intron of the transfrag overlaps a reference intron on the opposite strand(likely due to read mapping errors; u: Unknown, intergenic transcript; x: Exonic overlap with reference on the opposite strand. The x, i, j, u, and o groups represented the potential new transcripts and the genes of u group were new genes.
